# Artificial Intelligence Based Customer Churn Prediction Model for Business Markets

**DOI:** 10.1155/2022/1703696

**Published:** 2022-09-29

**Authors:** J. Faritha Banu, S. Neelakandan, B. T Geetha, V. Selvalakshmi, A. Umadevi, Eric Ofori Martinson

**Affiliations:** ^1^Department of Computer Science and Engineering, SRM Institute of Science and Technology, Ramapuram, Chennai, India; ^2^Department of CSE, R.M.K Engineering College, Chennai, India; ^3^Department of ECE, Saveetha School of Engineering, SIMATS, Saveetha University, Thiruvallur, India; ^4^Department of Management Studies, SRM Valliammai Engineering College, Kattankulathur, Tamilnadu, India; ^5^Department of Electronics and Communications Engineering, School of Engineering, All Nations University, Koforidua, India

## Abstract

The introduction of artificial intelligence (AI) and machine learning (ML) technologies in recent years has resulted in improved company performance. Customer churn forecast is a difficult problem in many corporate sectors, particularly the telecommunications industry. Because customer churns have a direct impact on a company's total revenue, telecommunications firms have begun to develop 76 models to reduce churns at an earlier stage. Previous research has revealed that AI and ML models are effective CCP solutions. According to this viewpoint, this study proposes a unique AI-based CCP model for Telecommunication Business Markets (AICCP-TBM). The AICCP-TBM model's purpose is to control the existence of churners and non-churners in the telecom sector. The proposed AICCP-TBM model employs a Chaotic Salp Swarm Optimization-based Feature Selection (CSSO-FS) method for the best feature assortment. In addition, a Fuzzy Rule-based Classifier(FRC) is used to distinguish between client churners and non-churners. A technique known as Quantum Behaved Particle Swarm Optimization (QPSO) is used to pick the membership functions for the FRC model in order to improve the classification performance of the FRC model. The performance of the AICCP-TBM model is validated using a benchmark CCP dataset and the experimental results are reviewed from several angles. In relations of presentation, the imitation consequences demonstrated that the AICCP-TBM model surpassed the most recent state-of-the-art CPP models. The suggested AICCP-TBM method's comparative accuracy was thoroughly tested on the three datasets used. Using datasets 1-3, this technique obtained better levels of accuracy, with the maximum attainable values being 97.25 %, 97.5 % and 94.33 %. The simulation results for the AICCP-TBM model demonstrated improved prediction performance.

## 1. Introduction

**Figure 1:**
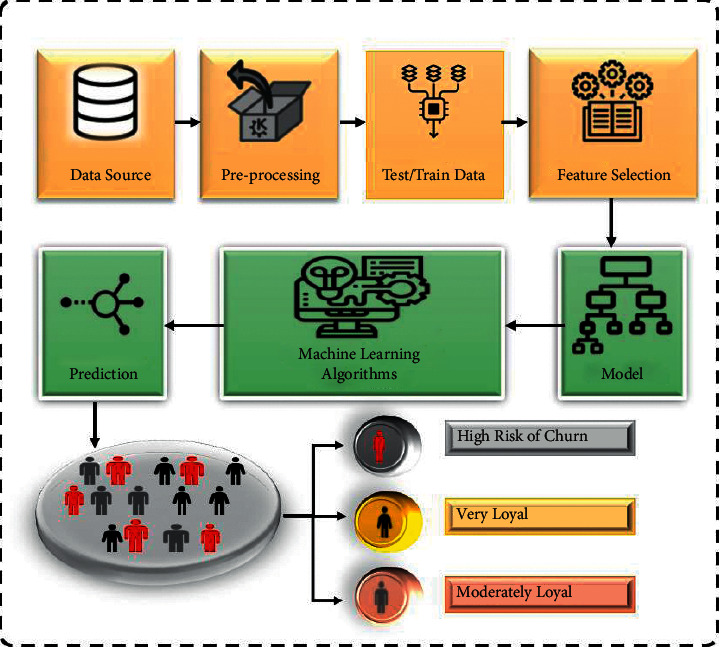
General Framework of CCP

While introducing the quarter manufacturing revolution, Machine Learning (ML) and Artificial Intelligence (AI) methods are driving corporate automation in a variety of areas, from forecasting optimal transport loads to shortlisting loan candidates without the need for human intervention [1]. Machine learning (ML) is a subset of artificial intelligence (AI) that allows software applications to enhance their prediction abilities without having to be explicitly programmed to do so. It is becoming increasingly popular. Machine learning algorithms anticipate future output standards using historical data. Machine learning is an artificial intelligence subfield that can be broadly defined as a machine's ability to emulate intelligent human behaviour. Artificial intelligence systems are used to perform complex tasks in the same way that people do. This technology promises to be more expensive than humans, but it may also be difficult. Automated trading strategies, in particular, have produced showy bangs in the US stock market [2] and one of Uber's self-driving vehicles hit and killed a ordinary. AI systems could use guidelines, learn in real time by acquiring new information and data (i.e., via ML) and adapt to changes in their platform. While AI applications are widely used in businesses, they generally consist of three parts (Figure 1). Input data is a starting component that is required for AI to work; without it, AI is characterised as a numerical fiction. AI has the ability to manage massive amounts of data, making it extremely important in the advent of big data. Furthermore, AI is capable of utilising both unstructured inputs such as conversations/speech and images, as well as structured inputs such as data transactions [3]. Several businesses use historical data in their AI applications. Fraudster, in particular, detects payment fraudulence by analysing data interactions with IP connection type, shipping and billing addresses. Furthermore, AI might make use of data collected in the actual world, whether through physical sensors or by tracking internet activities. The primary AI components are then the ML approach, which is the calculation process that processes the data input. There are tierce types of ML methods: reinforcement, oversaw and unverified. Machine learning (ML) is a subset of artificial intelligence (AI) that enables software programmes to enhance their prediction skills even when they were not explicitly designed to do so. Machine learning algorithms anticipate future output values using previous data. Machine learning is an artificial intelligence subfield that may be generally defined as a machine's capacity to emulate intelligent human behaviour. Artificial intelligence systems are used to do complex tasks in the same way that people do. In supervised ML, a human expert provides the computer with training datasets containing inputs and outputs so that the process can learn patterns and improve guidelines for future occurrences of comparable difficulties. Supervised learning occurs when all of the observations in a dataset are branded and the procedures learn to forecast the output from the input information as they learn to learn from the input data. Unsupervised learning: All of the observations in the dataset are unlabelled and the algorithms learn from the input data to recognise intrinsic structure in the data. Reinforcement The process of reinforcing models in order to understand how to make decisions is known as learning. This type of learning is extremely fascinating to analyse and it is one of the most widely researched subjects in machine learning. When utilising this method, the algorithm assists in causing the model to learn based on the feedback it receives. AI could be trained specifically to detect tiny cell changes in Magnetic Resonance Imaging (MRI) scans in order to detect cancer at an earlier stage [4]. The output decision from the ML algorithm is the third major AI component. At the bottom of the continuum, AI may produce a single result, such as a deception score [5], which has no performable values until a predictor decides to perform on it. For telecom clients, an AICCP-TBM model like this one is important. Verbeke et al. (2012). By making computers easy to operate, this improves churn detection. Using CSSO-FS algorithms, it finds the best feature subsets from previously processed data. Overall prediction performance improves when the QPSO algorithm is employed to classify churners in telecommunications. CSSO and QPSO are the best algorithms. There is no other algorithm that can compete.

### 1.1. Significance of CCP in Telecom Markets

In developed countries, the telecommunications sector must become one of the major sectors. The level of competition has risen as a result of technology advancements and an increase in the number of operators [6]. Telecommunications is a key tool for businesses. It enables organisations to efficiently communicate with customers and provide outstanding customer service. Telecommunications is a vital component in enabling employees to efficiently interact from any place, whether remote or local. Corporations are working hard to survive in this modest marketplace using a variety of strategies. For generating extra revenues, three basic ways have been proposed: (1) acquiring new consumers, (2) upselling existing customers and (3) increasing client retention. It is possible to calculate the efficiency or profitability of an investment using a return on investment (ROI) statistic, and to compare the efficiency of multiple investments in order to determine which is the most efficient. Return on investment (ROI) is a performance metric that can be used to determine the efficiency or profitability of an investment, as well as to determine which investment is the most efficient. The term “rate of return on investment” (ROI) refers to the rate of return on a specific investment in relation to the amount of money invested. The following is the formula for determining return on investment (ROI):

Return on Investment (ROI) = Current Investment Value minus Cost of Asset is calculated by dividing the price of asset by the cost of investment.

The term “Current Value of Investment” refers to the amount of money received upon the sale of an interest-bearing investment at the time of the sale. It is simple to compare a return on investment (ROI) to the returns on other investments since the % is expressed as a percentage. This makes it possible to compare returns across a variety of various investment types. However, when relating this technique, the value of Return on Investment (RoI) of each consideration is considered. has demonstrated that the third strategy is more cost-efficient method, demonstrating that sustaining an existing customer cost less than acquiring a new one, as well as being significantly easier than the upselling approach. Companies should reduce the risk of client churn, also known as “the customer movement from one supplier to another,” in order to utilise the third technique. The use of data analysis to forecast churn aims to predict if an individual customer will churn, the timeframe for business and the reasons for churn. Telecommunications companies can reduce churn by forecasting which customers are most likely to depart and offering them alternative and better incentives or packages to keep them. Many scholars have successfully addressed the churn prediction topic using a range of machine-learning techniques in addition to data mining methodologies. It is safe to assume that churn prediction is one of data science's most important commercial applications. The fact that its effects are more tangible and play a substantial influence in the company's total revenues is what makes it so popular with business owners and executives. The issues surrounding Customer Churn Prediction (CCP)- The loss or outflow of regulars from a company's client base is characterised as a customer's churn rate, also known as customer attrition or customer defection. In oversaturated markets, there are few opportunities for expansion, or significant investments are required to attract new clientele. Figure 1 depicts the general framework of CCP.

The data mining technology makes it easier to predict a customer's future behaviour. Customer churn, also known as client attrition, is one of the primary matters that decreases a corporation's revenue [7]. Customer churn, customer attrition and customer defection are all terms used to describe customer attrition. The churn rate, also known as attrition or customer churn, is the rate at which a company's customers decide to stop doing business with the organisation. It is typically represented as a proportion of facility contributors who terminate their memberships within a given time frame. It is also the degree at which people abandon their occupations after a convinced retro of time. A company's growth rate (as unhurried by the quantity of new customers) must surpass its attrition rate in order to expand its customer base. In today's businesses, a plethora of options enable clients to benefit from a competitive market. One could choose a service provider who provides the best service in comparison to others. Thus, profit-making enterprises fight in saturated industries such as internet service providers, banks, telecommunications and insurance companies, with a focus on maintaining current customers rather than acquiring new consumers. Furthermore, retaining current customers has been shown to be less expensive than getting new clients [8].

In order to keep clients, businesses must first understand why they leave. There are several difficulties to solve, such as competitive pricing from other firms, customer relocation, dissatisfaction with the company and the client's reliance on outstanding service, all of which could lead clients to abandon their present facility worker and go to another. Among previous studies for churn analysis, the most widely used technique is Artificial Neural Networks (ANNs). Many methodologies and topologies have been investigated employing ANNs for fine tuning the established modules, such as developing medium-sized ∗methods that are seen to execute an optimal and make investigations on numerous fields such as finance, pay-TV, retail and banking [9]. There is a great deal of attention in employing Artificial Neural Networks (ANNs) to solve tough problems such as churn prediction. Convolutional, convolutional neural and recurrent neural networks are only a few of the topologies and learning methods that can be used by computer models or hardware-based neural networks. A typical supervised model, the Multi-Layer Perceptron, is trained using Back-Propagation Algorithm modifications (BPN). The BPN's feed-forward design is based on supervised learning. Au and colleagues show that neuronal networks outperform Decision Trees(DT) in the problem of customer attrition. ANN outperformed Logistic Regression and C5.0 in forecasting customer churn. Logistic Regression (LR) is a statistical classification model in probability. For example, customer churn can be forecasted binary utilising one or more predictor factors (e.g., customer characteristics). After sufficient data processing, LR is commonly used for churn prediction and it performs as well as, if not better than, DT.

### 1.2. Paper Contributions

This study provides an AI-based Customer Churn Prediction (CCP) model for telecommunications business markets (AICCP-TBM). The importance of the report is that it would help telecom corporations estimate customer attrition and increase revenue. The AICCP-TBM model has several stages of operations, including pre-processing, feature selection, classification and parameter optimization. Furthermore, the proposed AICCP-TBM model classifies churners and non-churners using a Chaotic Salp Swarm Optimization-based Feature Selection (CSSO-FS) technique and a fuzzy rule-based classifier (FRC). The Quantum Behaved Particle Swarm Optimization (QPSO) algorithm is also used to determine the FRC technique's Membership Functions (MF). The quantum behaving particle swarm approach is an innovative intelligent optimization strategy with few parameters and a straightforward implementation. The experimental results show that the changed algorithm improves the method's capacity to optimise. In computing, the quantum particle swarm optimization approach is a programme that ensures worldwide convergence of calculations. Based on a unique learning strategy that blends cross-sequential quadratic programming with Gaussian chaotic mutation operators, this technique can be used to learn new skills. In order to evaluate the increased prediction performance, a complete investigational examination is achieved and the consequences are evaluated in terms of numerous factors. The following is a summary of the contributions to the papers.

- A new AICCP-TBM model for CCP in the telecom sector is offered, which incorporates pre-processing, CSSA-based feature selection and QPSO-FRC-based classification. The AICCP-TBM model has not been constructed in any previous studies that the author is aware of.
- Creates a CSSA technique for feature selection by including chaotic maps into the classic SSA and changing the random parameters, hence increasing the convergence rate.
- Proposes a QPSO-FRC classification technique in which the FRC model's MF is efficiently selected using the QPSO algorithm. It aids in the accurate classification of previously unseen customer churn data.

### 1.3. Paper Organization

According to the following organisational framework, the remainder of the paper will be written. The second section contains a summary of the most recent CCP models that have been published.  Section 3 describes the AICCP-TBM model, while Section 4 describes the simulation process.  Section 3 describes the AICCP-TBM model.  Section 5 concludes the investigation and brings it to an end.

## 2. Existing CCP Models for Telecommunication Sector

Ahmad et al. [10] created a churn prediction system that assists telecom operators in forecasting clients who are likely to churn. This study's strategy employs ML methods in a large data context to build a novel method of feature selection and engineering. Other significant advances include the use of client social networks in the prediction method through the extraction of SNA properties. This approach was evaluated and developed by the Spark platform by running it on a huge dataset shaped by altering large raw data providing by SyriaTel Telecom Corporation. Two ML approaches were used by Jain et al. [11] to predict CC Logit Boost and LR. The study was carried out using WEKA ML equipment and an actual database from the American corporation Orange.

Saravana Kumar et al. [12] suggested a CCP technique that employs soft voting and SACtacking models via an ensemble learning method. Machine learning methods Xgboost, LR, DT and NB are utilised to build a two-stage stacking approach, with the three outputs of the succeeding level used for soft voting. Machine learning algorithms make predictions about future output values based on prior data analysis. An artificial intelligence area known as machine learning is described as the ability of a machine to reproduce intelligent human behaviour in a wide sense. Artificial intelligence systems are utilised to do complicated tasks in a manner that is similar to that of a human. In Vijaya and Sivasankar [13], RST presents a method for identifying the most successful aspects of telecommunication CCP. The selected characteristics are then supplied into ensemble classification methods like Random Subspace, Bagging and Boosting. In this study, the efficiency of the Duke University churn predictive datasets is assessed using three unique sets of investigations. RB [14] develops and designs the Fine-tuned XGBoost method, which overcomes the problems of imbalanced datasets by presenting the feature function; it also handles the concerns of overfitting and data sparsity.

The use of data analysis to forecast churn aims to predict if an individual customer will churn, the timeframe for turnover, and the reasons for churn. Telecommunications companies may minimise churn by forecasting which customers are most likely to depart and providing various and better incentives or packages to entice them to stay. Many scholars have successfully addressed the churn prediction topic using a range of machine-learning techniques in addition to data mining methodologies. It is reasonable to assume that churn prediction is one of data science's most important business applications. The fact that its effects are more tangible and play a substantial influence in the company's total revenues is what makes it so popular with business owners and executives.

Mohammad et al. [15] sought to identify the factors that influence CC, build an effective churn prediction system and give an optimal analysis of data visualisation results. The dataset was obtained from the open data website Kaggle. The proposed approach for assessing churn prediction involves a number of processes, including data pre-treatment, analysis, the use of ML algorithms, the computation of the classifier and the selection of the best one for forecasting. The data pre-processing procedure consists of three major steps: feature selection, data cleaning and data transformation. For LR, ANN and RF, the ML classifier is used. Al-Mashraie et al. [16] compare the efficacy of various churn forecasting algorithms using real data obtained from a companion corporation. Furthermore, the PPM design is utilised to investigate the impact of mooring, push and pull perceptions on CC behaviour. The PPM analysis are carried out using a PLS regression. The behaviour of churners and non-churners is also investigated.

De Caigny et al. [17] look into the value added by merging textual data with CCP techniques. It extends the previous study, which used a traditional CNN, to current ideal practises for textual data analysis in CCP and verifies an architecture for textual data analysis in CCP using real-world data from a European monetary services organisation.

In an open-source Telecoms dataset, Halibas et al. [18] performed exploratory data analytics and feature engineering, employing 7 classification methods such as Naïve Bayes(NB), Generalized Linear Model, LR, Deep Learning(DL), DT, RF and Gradient Boosted Tree(GBT). Different measurements are used to evaluate the results, including AUC, Accuracy, Precision, Classification error, Recall and F1-score. The churn rate, also known as attrition or customer churn, is the rate at which a company's customers decide to stop doing business with the organisation. A percentage of service subscribers that terminate their memberships within a particular time period is occasionally used to represent this figure. Additionally, it refers to the rate at which employees quit their jobs after a given period of time has elapsed [19]. The churn rate, also known as attrition or customer churn, is the rate at which a company's customers decide to stop doing business with it. It is measured in percentages. This metric is sometimes represented as a percentage of service subscribers who terminate their memberships within a specific length of time, as in the case of Netflix. Aside from that, it refers to the rate at which employees quit their jobs after a specific amount of time has passed. A unique profit-centric performance metric is developed as a result of the first element of this study's development of a unique profit-centric performance metric by estimating the maximum profit that can be produced by enrolling the optimal proportion of customers with the highest projected attrition rate in a retention campaign [20]. When compared to statistical alternatives, the unique metric indicates the ideal model to use and the optimal customer fraction to include, resulting in a significant increase in revenue.

## 3. The Proposed AICCP-TBM Model

**Figure 2:**
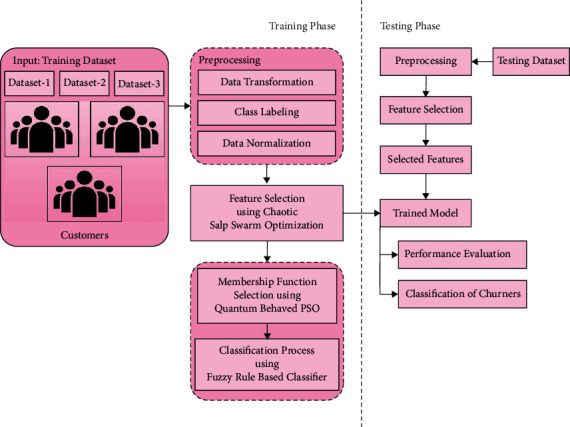
Overall process of AICCP-TBM model

In order to build the AICCP-TBM model, a set of processes are involved as shown in Figure 2. A detailed explanation of these modules is offered in the following units.

### 3.1. Data Pre-processing

During data pre-processing, customer data is pre-processed in three steps: data transformation, class labelling and data normalisation. Second, instances are assigned to relevant classes during the class labelling process. In this knowledge normalisation technique, the initial data is transformed using a linear transformation. The data's minimum and maximum values are extracted, and each value is replaced using the formula below. Min-Max During normalisation, the relationships between the original data values are kept [21]. An out-of-bounds error will occur if a subsequent input case for normalisation exceeds the first data range for x. Third, data normalisation is carried out using the min-max dataset, as shown below:

(1) $$\begin{array}{c} \mathrm{Min}-\mathrm{Max}.Norm=\frac{x-x_{\min}}{x_{\max}-x_{\min}}\text{.} \end{array}$$

### 3.2. Algorithmic Design of CSSO-FS Technique

Aside from reprocessing data, the CSSO-FS technique seeks to select an optimal subset of attributes from a large amount of data. Swarms of salps serve as inspiration for the Salp Swarm Algorithm (SSA), which is a revolutionary bioinspired algorithm. CSSO is an optimization strategy that makes use of chaotic variables rather than random variables to achieve a desired result. CSSO uses chaotic maps to change the value of a parameter. In order to increase the convergence of the PSO method, chaotic maps are used instead of random numbers [22]. In order to improve the performance of meta-heuristic algorithms, raising the randomization settings on chaotic maps makes them the most powerful method for doing so. The use of chaotic maps to regulate these parameters reduces the number of local optima and increases the speed with which they are reached. The new CSSO approach is introduced, in which chaotic maps are utilised to substitute disordered SSA parameters for arbitrary parameters in a random number generator. With regard to the SSA population, there are two distinct subgroups: leaders and followers. The former is referred to as the chain's leader, and the remaining of the salps are referred to as followers [23]. Consider the variables dim, which shows the number of dimensions, y, which indicates the location of the salp, and F, which reflects the availability of food. In addition, (2) can be used to update the location of the leader in a given situation.

(2) $$\begin{array}{c} y_{i}^{1}=\left\{\begin{array}{cc} F_{i}+r_{1}\left(\left(ub_{i}-lb_{i}\right)r_{2}+lb_{i}\right)\text{,} & r_{3}\geq 0\text{,}\\ F_{i}-r_{1}\left(\left(ub_{i}-lb_{i}\right)r_{2}+lb_{i}\right)\text{,} & r_{3}<0\text{.} \end{array}\right. \end{array}$$

The followers can update the position based on Newton's law of wave in (3). Here, *y*_*i*_^*j*^ represents the place of j-th followers in ith dimensional, *i* ≥ 2, *β*_0_ implies the first speed, *α*=*β*_final_/*β*_0_, *β*=*y* − *y*_0_/*t* and *l* is time. Thus, the time is named as iteration in optimizes procedure, the discrepancy in the iteration equivalent to one. Assume *β*_0_=0, the upgrading place of followers in ith dimensional is signified as:

(3) $$\begin{array}{c} y_{i}^{j}=\frac{1}{2}\left(y_{i}^{j}+y_{i}^{j-1}\right)\text{.} \end{array}$$

There are 3 major variables that influence its efficiency as are *r*_1_,*r*_2_*andr*_3_. As displayed in (2), *r*_1_ is reduced linearly by the iterations, where *r*_3_ is accountable to determine either the following location has to be towards positive/negative infinity. Since it is shown in (2), *r*_2_ & *r*_1_ represents the 2 major variables affecting the upgrading location of a salp[18]. As a result, they have a significant impact on the balance of exploitation and exploration. Research is concerned with identifying new, optimal solutions by extensively researching the entire search area, whereas exploitation is concerned with making use of the information in the local neighbourhood. A technique must appropriately balance these two aspects in order to come near to the global ideal. In this work, chaotic map is utilized for adjusting a *r*_2_ variables of SSA. Eq. (4) displays the upgrading of *r*_2_ variable as per the chaotic map. Eq. (4) displays the upgraded location of a salp as per the chaotic map, whereas *o*^*t*^ implies the attained value of chaotic map in *t*-*th* iteration.

(4) $$\begin{array}{c} r_{2}=o_{s}\text{,} \end{array}$$

(5) $$\begin{array}{c} y_{i}^{t}=\left\{\begin{array}{cc} F_{i}+r_{1}\left(\left(ub_{i}-lb_{i}\right)o^{t}+lb_{i}\right)\text{,} & r_{3}\geq 0\text{,}\\ F_{i}-r_{1}\left(\left(ub_{i}-lb_{i}\right)o^{t}+lb_{i}\right)\text{,} & r_{3}<0\text{.} \end{array}\right. \end{array}$$

The goal of this embedding chaotic map, as stated in the following section, is to update the location of salp, which may increase the convergence rate and performance of SSA. The CSSO-FS methodology confined the solution pool for separating the binary method, where the position of salps is constrained to zero and one. Here, a salp location (solution in the search space) *y* formulated as *i*−*th*‐dimension parameter *y*=[*x*_1_, *x*_2_,…, *x*_di m_], whereas di m denotes the maximal quantity of scopes[24–27]. It is chosen the consistent characteristics when the value of the parameter equals one; when the value of the parameter equals zero, it is decided not to choose the equivalent features. More precisely, the data's feature representation contains five characteristics as 1, 0,0, 1, 1 or 0, 1 , 1, 1 etc. It is clear from the last two examples that all of the solutions have distinct features and all of the solutions have distinct lengths. (6) shows all of the agents' transition from continuous to discrete binary space, where B represents an arbitrary quantity between zero and one.

(6) $$\begin{array}{c} y_{i}^{t}=\left\{\begin{array}{cc} 1\text{,} & if\left(s\left(y_{i}^{t}\right)\right)\geq B\text{,}\\ 0\text{,} & else\text{,} \end{array}\right. \end{array}$$

(7) $$\begin{array}{c} s\left(y_{i}^{t}\right)=\frac{1}{1+e^{10\left(y_{i}^{t}-0.5\right)}}\text{.} \end{array}$$

Then, the complete descriptions of the presented chaotic form of SSA method are given below: ‐

#### 3.2.1. Parameter initialization

To begin, the CSSO assumes a salps position that has been arbitrarily initiated. Following that, it sets the initial values for the major variables. The lower and upper bounds of the standard function used are present at the outset, but the lower and upper bounds of the standard dataset used are largely set to zero and one for the data that is provided. For solving global optimization problems, the maximum number of iterations is 500, but the maximum number of iterations when solving FS problems is thirty. Finally, the population size for global optimization problems is set to fifty, whereas the population size for FS problems is set to twenty[28–31]. Initially, the search agents are assigned in a random manner. The upper and lower bounds of the global benchmark functions are set to [Math Processing Error] for the data that has been submitted. Errors in global optimization as well as feature selection This is owing to the fact that the search space is quite complex. The [Math Processing Error] global element is the most useful. While the total number of global optimizations is limited to 500, the total number of FS optimizations is limited to 30 in this case. At the end, the population size is reduced to fifty for global optimization and twenty for finite state optimization. CSSO is an optimization strategy that makes use of chaotic variables rather than random variables to achieve a desired result. With the use of chaotic maps, CSSO can change the value of a parameter. In order to increase the convergence of the PSO method, chaotic maps are used instead of random numbers. In order to improve the performance of meta-heuristic algorithms, raising the randomization settings on chaotic maps makes them the most powerful method for doing so. Regulatory these limits with chaotic maps reduces the number of local optima and upsurges meeting.

#### 3.2.2. Fitness function (FF)

It is used to assess the full set of solutions (salp positions). Each global standard problem that is used is a minimization problem. As a result, the explanation with the lowest suitability value is chosen as the best choice available thus far. In contrast, the best technique for FS tasks maximises organization correctness while minimising the quantity of features picked. (8) depicts the FF utilised to solve FS problems, whereas specifying a weight factor combines these two goals into one. In this formula, signifies the classification accuracy gained using FRC classification, whereas The goals of this work are to first enhance classification accuracy and then to reduce the number of features chosen[32]. Therefore, *w*_*f*_ is fixed to 0.8. *L*_*f*_ indicates the length of features selected subset, whereas *L*_*t*_ represents the overall amount of features for a provided dataset.

(8) $$\begin{array}{c} Fn_{t}=maximize\left(w_{f}\times Acc+\left(1-w_{f}\right)\times \left(1-\frac{L_{f}}{L_{t}}\right)\right)\text{.} \end{array}$$

#### 3.2.3. Positions updating

Later calculating the FF of every salp, choose an optimal salp location. The optimal salp informs its positions as per (5), (6), (2) and (3).

#### 3.2.4. Termination criteria

**Figure 3:**
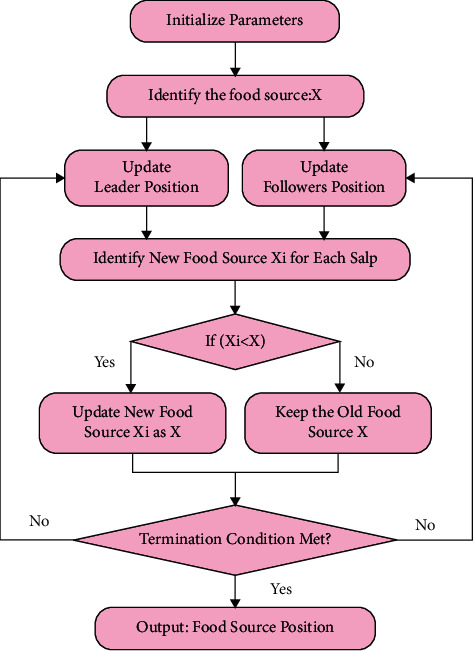
Flowchart of SSA

The development of calculating each salp and improving the location of the ideal salp would be performed continuously until the extreme number of repetitions was reached or the best solution was determined. In this study, the optimization technique stops when the extreme sum of iterations is reached; fifty for worldwide optimization problems and thirty for FS problems[33]. The flow chart of SSA is shown in Figure 3 and the stages are given in Algorithm 1.

### 3.3. Data Classification using QPSO-FRC Technique

**Figure 4:**
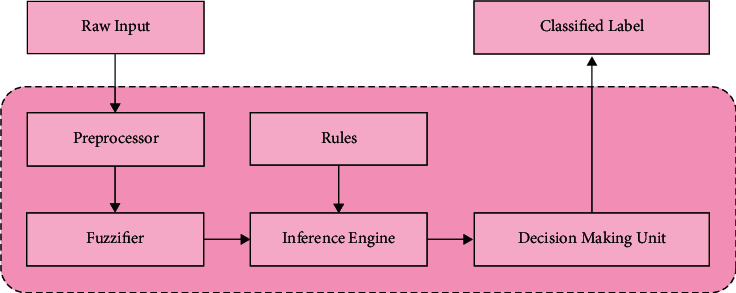
Structure of FRC

In the last stage, the QPSO-FRC approach is used to divide customers into two groups: churners and non-churners. To distinguish between client churners and non-churners, a fuzzy rule-based classifier is also used (FRC). The Quantum Behaved Particle Swarm Optimization (QPSO) technique is used to determine the membership functions in order to improve the classification performance of the FRC model. The FRC technique is a rule-based strategy that has significant benefits based on its design functionality and subsequent assessments [34]. The interpretability of classification rules is a exclusive benefit of uncertain classifiers. Assume that *x*=(*x*_1_, *x*_2_,…, *x*_*D*_) ∈ *R*^*D*^ denotes *D*‐dimension feature space and *C*={*c*_1_, *c*_2_, ..., *c*_*m*_} represents a group of class labels. Later, the classification difficulties could be abridged to determining a tag that is equivalent to the feature course of an item to be categorised from a list of class labels. Fuzzy Rule-Based Classification Systems (FRBCSs) are a general design credit and machine learning tool. Because of the use of language labels in their rule antecedents, these systems exhibit excellent performance while also providing interpretable models. The structure of the FRC model is depicted in Figure 4. A fuzzy classifier is provided using the following production rules:

(9) $$\begin{array}{c} R_{i}:IFs_{1}\wedge x_{1}\text{,}\\ A_{1i}\wedge s_{2}\wedge x_{2}=A_{2i}\wedge \ldots \wedge s_{D}\wedge x_{D}=A_{Di}THENclass=c_{i},i=1,\ldots ,R\text{,} \end{array}$$

whereas *A*_*ki*_ denotes the uncertain term which illustrates the *k*^th^ feature in *i*^th^ fuzzy rule (*k*=1,…, *D*), *R* indicates the quantity of uncertain rules and *S*=(*s*_1_, *s*_2_,…, *s*_*D*_) represents the binary feature vector, whereas *s*_*k*_∧*x*_*k*_ denotes the presence (*s*_*k*_=1) or absence (*s*_*k*_=0) of feature in the classifier [22]. In a provided dataset {(*x*_*p*_; *c*_*p*_), *p*=1,2,…, *Z*} the class label is determined by:

(10) $$\begin{array}{c} class=c_{t},t=arg\max_{j=1,2,\ldots ,m}\beta_{j}\text{,}\\ \beta_{j}\left(x_{p}\right)=\underset{\begin{array}{c} R_{i}\\ class_{i}=c_{j} \end{array}}{\sum }\overset{D}{\underset{k=1}{\prod }}{µ}_{A_{ki}}\left(x_{pk}\right)\text{,} \end{array}$$

*µ*_*A*_*ki*__(*x*_*pk*_) denotes the symmetric MF for the uncertain term *A*_*ki*_ at point *x*_*pk*_.

The organization rate is calculated as the ratio of the amount of correctly allocated class labels to the total sum of substances to be categorised:

(11) $$\begin{array}{c} E\left(\theta ,S\right)=\frac{\sum_{p=1}^{Z}\left\{\begin{array}{cc} 1, & ifc_{p}=arg\max_{j=1,2,\ldots ,m}f_{j}\left(x_{p};\theta ,S\right),\\ 0, & otherwise \end{array}\right.}{Z}\text{,} \end{array}$$

Whereas *f*(*x*_*p*_; *θ*, *S*) means the production of uncertain classifier using the limit *θ* and feature *S* at the point *x*_*p*_.

For improving the performance of the FRC technique, the MFs are chosen by the QPSO algorithm. In the novel PSO, all the particles are determined using a location vector *x*=(*x*_1_, *x*_2_,…, *x*_*D*_) that indicates solution in the search space and related to velocity vector *v*=(*v*_1_, *v*_2_,…, *v*_*D*_) accountable to the exploration of search space. Here, *N* denotes swarm size and *D* dimension of the search space, in evolution procedure, the velocity and the location of all particles are upgraded by:

(12) $$\begin{array}{c} V_{i,d}^{t+1}{\omega v}_{i,d}^{t}+c_{1}r_{i,d}^{t}\left(pbest_{i,d}^{t}-x_{i,d}^{t}\right)+c_{2}R_{i,d}^{t}\left(gbest_{d}^{t}-x_{i,d}^{t}\right)\\ x_{i,d}^{t+1}=x_{i,d}^{t}+v_{i,d}^{t+1}\text{,} \end{array}$$

whereas *i*(1 ≤ *i* ≤ *N*) & *d*(1 ≤ *d* ≤ *D*),*v*_*i*,*d*_^*t*^ & *x*_*i*,*d*_^*t*^ denotes *d*th dimension element of velocity and location of particles *i* in search iteration *t*, correspondingly, *p*best_*i*,*d*_^*t*^ & *g*best_*d*_^*t*^ denotes *d*th dimension of individual optimum of particles *i* and global optimal of swarm in search iteration *t*, correspondingly, *v* denotes inertia weight, *c*_1_ & *c*_2_ represented 2 positive constant acceleration coefficients and *r*_*i* *d*_^*t*^ and *R*_*i*,*d*_^*t*^ denotes 2 arbitrary numbers of uniform distribution in the range of zero and one[27]. As per the trajectory analyses, the convergence of the PSO method might be attained when the entire particles converge to its local attractor *p*_*i*_=(*p*_*i*,1_, *p*_*i*,2_,…, *p*_*i*,*D*_), coordinate is determined by

(13) $$\begin{array}{c} p_{i,d}^{t}=\phi_{d}^{t}\times pbest_{i,d}^{t}+\left(1-\phi_{d}^{t}\right)\times gbest_{d}^{t}\text{,} \end{array}$$

where *ϕ*_*d*_^*t*^=*c*_1_*r*_*i*,*d*_^*t*^/(*c*_1_*r*_*i*,*d*_^*t*^+*c*_2_*R*_*i*,*d*_^*t*^).

The idea of QPSO was established depending upon the aforementioned analyses. All the individual particle in QPSO is preserved as a rotation less one moves in important space and the likelihood of particle is appear at the location *x*_*i*_^*t*^ in search iteration, *t* is defined after a likelihood density purpose [35]. Employ the Monte Carlo technique, all the particles fly by:

(14) $$\begin{array}{c} x_{i,d}^{t+1}=p_{i,d}^{t}+\alpha \vee x_{i,d}^{t}-mbest_{d}^{t}\vee \ln\left(\frac{1}{u_{i,d}^{t}}\right),ifrandv\geq 0.5\text{,}\\ x_{i,d}^{t+1}=p_{i,d}^{t}-\alpha \vee x_{i,d}^{t}-mbest_{d}^{t}\vee \ln\left(\frac{1}{u_{i,d}^{t}}\right),ifrandv<0.5\text{,} \end{array}$$

Whereas *α* denotes variable named contraction expansion coefficient; *u*_*i*,*d*_^*t*^ ∗ ran dv denotes arbitrary number of uniform distributions between zero and one; mbest denotes global virtual point named mainstream or mean optimal determined by

(15) $$\begin{array}{c} mbest_{d}^{t}=\frac{1}{N}\overset{N}{\underset{i=1}{\sum }}pafbest_{i,d}^{t}\text{.} \end{array}$$

Typically, a time-varying reduction approach is used to adjust the contraction expansion coefficient by:

(16) $$\begin{array}{c} \alpha =\alpha_{1}+\frac{\left(T-t\right)\times \left(\alpha_{0}-\alpha_{1}\right)}{T}\text{,} \end{array}$$

where *α*_0_ & *α*_1_ denotes first and last value of *α*, correspondingly; *T* denotes maximal amount of iterations; *t* indicates the present search repetition quantity. The QPSO approach, which has simpler evolution equations and fewer parameters than classic PSO, greatly simplifies convergence and control in the search space. The process for implementing the QPSO is presented with no loss of generality, where *f* signifies the objective function to be decreased.

## 4. Performance Validation

### 4.1. Dataset Used

The accuracy of the AICCP-TBM approach in predicting three different CCP datasets is investigated in this section. If you want to understand more about the dataset, Table 1 is a fantastic place to start. Furthermore, the feature selection and predictive performance of the AICCP-TBM approach are studied across all three datasets.

### 4.2. Results and Discussion

**Figure 5:**
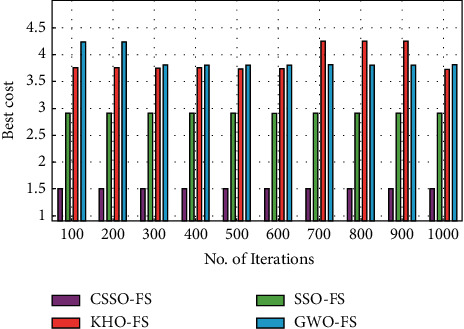
Best cost analysis of CSSO-FS model on dataset 1

Table 2 compares the CSSO-FS technique's cost analysis to other FS techniques on the three datasets examined.  Figure 5 depicts the CSSO-FS technique's best cost analysis in comparison to other FS approaches on the employed dataset 1. As shown in the image, the GWO-FS approach has clearly proved ineffective FS outcomes at the lowest possible cost. As a result, the KHO-FS technique has produced somewhat increased performance at a low cost. The SSO-FS technique, on the other hand, delivered a pretty reasonable performance at the lowest cost. In contrast, the CSSO-FS technique has produced effective outcomes at a reasonable cost. Based on the obtained data, the CSSO-FS strategy performs better with a lower regular best cost of 1.5002, whereas the SSO-FS, KHO-FS and GWO-FS techniques perform worse with higher average best costs of 2.9116, 3.8961 and 3.8929, respectively.

**Figure 6:**
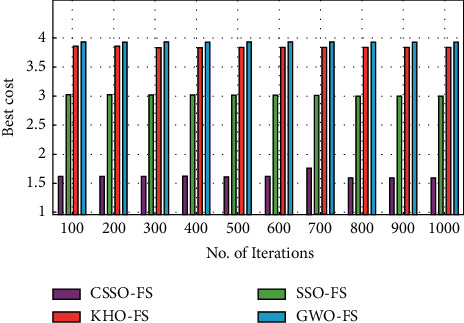
Best cost analysis of CSSO-FS model on dataset 2

Figure 6 depicts the CSSO-FS algorithm's best cost analysis in comparison to other FS methods on the used dataset 2. As seen in the figure, the GWO-FS approach produced ineffective FS outputs at the highest possible cost. Similarly, the KHO-FS technique offers somewhat improved performance despite having a moderate best cost. Furthermore, the SSO-FS approach yielded a marginally acceptable result at the lowest possible cost. Finally, the CSSO-FS approach yielded effective outcomes at the lowest possible cost. The CSSO-FS method performed better, with a minimum regular best cost of 1.6102, while the SSO-FS, KHO-FS and GWO-FS algorithms performed worse, with maximum average best costs of 3.0160, 3.8420 and 3.9320, respectively.

**Figure 7:**
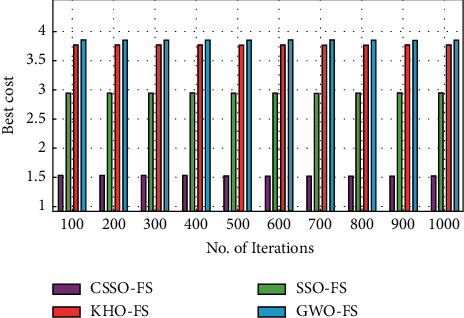
Best cost analysis of CSSO-FS model on dataset 3

Figure 7 depicts the CSSO-FS method's best cost analysis compared to other FS techniques on the employed dataset 3. As demonstrated in the figure, the GWO-FS technique produced ineffective FS results at the lowest cost. Similarly, the KHO-FS approach has produced somewhat higher efficiency with the moderate best cost. Furthermore, the SSO-FS approach produced a reasonably good result at the lowest possible cost. In addition, the CSSO-FS algorithm has provided effective outcomes at a reasonable cost. Based on the acquired figures, the CSSO-FS technique performs better with a lower regular best cost of 1.5250, but the SSO-FS, KHO-FS and GWO-FS methodologies perform worse with superior average best costs of 2.9481, 3.7748 and 3.8569, respectively.

**Figure 8:**
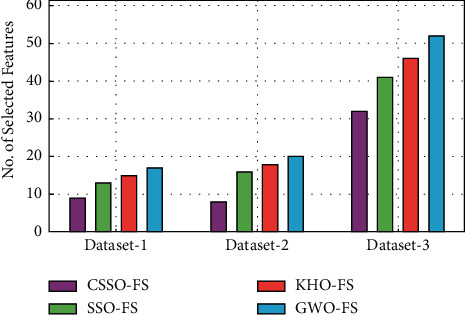
Feature selection analysis of CSSO-FS model

The number of features chosen by different FS techniques is computed in Table 3 and Figure 8 to further ensure FS performance. Based on the results, it is clear that the CSSO-FS methodology selected a small number of characteristics in comparison to other FS methodologies. For example, on the applied dataset-1, the CSSO-FS approach picked 9 features, but the SSO-FS, KHO-FS and GWO-FS techniques selected 13, 15 and 17 features, respectively.

Meanwhile, on the applied dataset-2, the CSSO-FS technique selected a minimum of 8 features, while the SSO-FS, KHO-FS and GWO-FS methods selected a maximum of 16, 18 and 20 features, respectively. Finally, on the applied dataset-3, the CSSO-FS approach picked 32 features, whilst the SSO-FS, KHO-FS and GWO-FS algorithms selected 17, 20 and 52 features, respectively.  Table 4 examines the prediction performance of the AICCP-TBM approach over a range of training sizes. The experimental results showed that the AICCP-TBM technique yielded beneficial results across a wide variety of training sizes. On the employed dataset-1, the AICCP-TBM technique revealed improved predictive outcomes, with an average compassion of 96.67 %, specificity of 97.33 %, accuracy of 97.26 % and F-score of 97.61 %. The AICCP-TBM technique produced enhanced prediction outcomes, with an average compassion of 96.92 %, specificity of 97.70 %, accuracy of 97.70 % and F-score of 97.70 %, according to the used dataset-2. Finally, the AICCP-TBM methodology outperformed enhanced prediction outcomes on the applied dataset-3 by an average of 95.88 % sensitivity, 94.77 % specificity, 94.33 % accuracy and 93.29 % F-score.

Finally, Table 5 [26, 27] presents a detailed comparison of the AICCP-TBM methodology with newly established methodologies. Based on the results, the SVM model has proven to be an inadequate performer when compared to the other approaches. Meanwhile, the WELM, PCPM and LDT/UDT approaches have shown marginally improved prediction results over the SVM model. Continuing, the SMOTE-OWELM and OWELM models produced reasonable predictive results on all datasets tested. Meanwhile, the SSO-FRBC and ISMOTE-OWELM models outperformed all of the other strategies save the AICCP-TBM technique. Among the compared approaches, the AICCP-TBM technique produced a proficient predictive result with highest accuracy of 97.25 %, 97.70 % and 94.33 % on the applied datasets 1-3. Founded on these consequences, it is clear that the AICCP-TBM technique can be used as an effective CCP tool in business markets, notably in the telecom sector.

**Figure 9:**
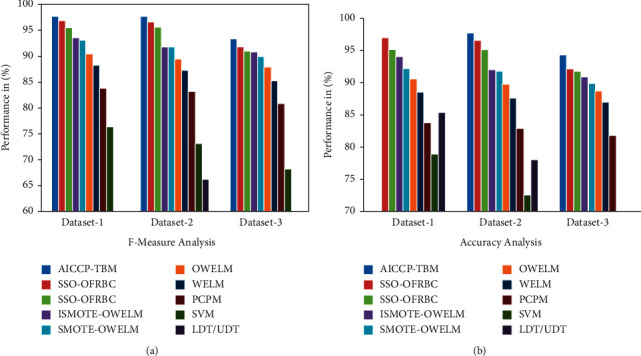
F-Measure analysis of proposed model with current methods Dataset-1, Dataset-2 and Dataset

Finally, Figure 9(a) and Figure 9(b) shows a thorough comparative accuracy and F-Measure analysis of the proposed AICCP-TBM technique on the three datasets used. On the applied datasets 1-3, the AICCP-TBM approach performed better, with maximum accuracy of 97.25 %, 97.70 % and 94.33 %.

## 5. Conclusion

An effective AICCP-TBM model is built in this study to determine churned/non-churned clients in the telecommunications sector. The AICCP-TBM model is intended to increase churn detection while requiring low computational complexity. The proposed AICCP model incorporates a CSSO-FS approach for selecting feature subsets from pre-processed customer data. Furthermore, the QPSO-FRC technique is employed in the telecoms industry for churner categorisation, where the usage of the QPSO algorithm for MF selection considerably enhances overall prediction performance. The inclusion of the CSSO and QPSO algorithms resulted in better projected results than previous techniques. To determine the effectiveness of the AICCP-TBM model, a full simulation study is performed on the benchmark CCP dataset. The simulation results indicated the increased prediction presentation of the AICCP-TBM perfect. When associated to the other techniques in this study, the SSO-FS, KHO-FS and GWO-FS approaches showed inferior performance and higher average best costs of 2.9116, 3.8961 and 3.8929, respectively. The SSO-FS method has the poorest presentation and the highest regular best costs of 3.8929, making it the most expensive technique. On the three datasets used, the proposed AICCP-TBM approach was submitted to a detailed comparative accuracy evaluation. The figure AICCP-TBM technique outperformed the other two techniques, with maximum accuracy of 97.25 %, 97.70 % and 94.33 % on the three datasets studied. The AICCP-TBM model outperformed the most recent state-of-the-art CPP models in rapports of performance, as indicated by the study's findings. In the upcoming, the future AICCP-TBM paradigm can be extended to a big data platform to handle real-time organisations' constant output of massive amounts of data. Big data analytics assists businesses in finding new clients. It's a simple equation: happy customers Equals Big Data. AI, Data Science and Deep Learning are frequently associated with Big Data. Big Data will be essential for improving current models and upcoming research. All strategies and tools for managing enormous datasets are referred to as big data. Big Data can assist businesses in identifying their most valuable customers. It can also help with the creation of new items. Big data can be used in marketing to obtain real-time cloud data. Big data analysis is useful since it saves time and money.

## Figures and Tables

**Algorithm 1:**
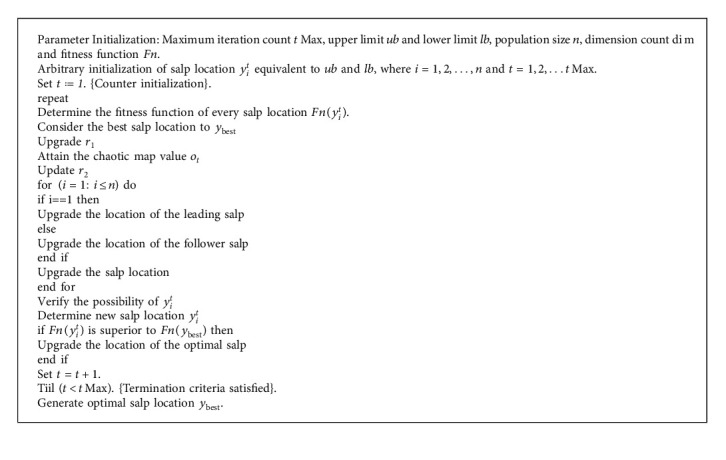


**Table 1:** Dataset Explanation

Description	Dataset-1	Dataset-2	Dataset-3
No. of Examples	3332	7042	100000
No. of Structures	20	20	100
No. of Class	3	3	3
%age of Positive Sample	14.48%	26.53%	49.55%
%age of Negative Sample	85.52%	73.45%	50.42%
Data source	[18]	[24]	[25]

**Table 2:** Result Analysis of Various Feature Selection methods on Applied Dataset

No. of Iterations	Dataset-1			
	CSSO-FS	SSO-FS	KHO-FS	GWO-FS
100	1.502	2.911	3.756	4.235
200	1.502	2.911	3.749	4.235
300	1.502	2.911	3.749	3.807
400	1.502	2.911	3.749	3.807
500	1.498	2.911	3.738	3.807
600	1.498	2.911	3.738	3.807
700	1.498	2.911	4.252	3.807
800	1.498	2.911	4.252	3.807
900	1.498	2.911	4.252	3.807
1000	1.498	2.911	3.721	3.807
**Average**	**1.500**	**2.911**	**3.896**	**3.892**

**No. of Iterations**	**Dataset-2**			
	**CSSO-FS**	**SSO-FS**	**KHO-FS**	**GWO-FS**
100	1.620	3.025	3.858	3.936
200	1.620	3.025	3.858	3.932
300	1.620	3.025	3.838	3.932
400	1.620	3.025	3.838	3.932
500	1.614	3.015	3.838	3.932
600	1.614	3.015	3.838	3.932
700	1.614	3.015	3.838	3.932
800	1.593	3.005	3.838	3.932
900	1.593	3.005	3.838	3.930
1000	1.591	3.005	3.838	3.930
**Average**	**1.610**	**3.016**	**3.842**	**3.932**

**No. of Iterations**	**Dataset-3**			
	**CSSO-FS**	**SSO-FS**	**KHO-FS**	**GWO-FS**
100	1.5325	2.9490	3.7770	3.8609
200	1.5325	2.9480	3.7770	3.8581
300	1.5325	2.9480	3.7770	3.8581
400	1.5325	2.9480	3.7770	3.8575
500	1.5216	2.9480	3.7750	3.8560
600	1.5216	2.9480	3.7730	3.8560
700	1.5194	2.9480	3.7730	3.8555
800	1.5194	2.9480	3.7730	3.8555
900	1.5191	2.9480	3.7730	3.8555
1000	1.5186	2.9480	3.7730	3.8555
**Average**	**1.5250**	**2.9481**	**3.7748**	**3.8569**

**Table 3:** Results of Number of Features Selected on Current with Proposed CSSO-FS Method on Applied Dataset

Methods	Dataset-1	Dataset-2	Dataset-3
CSSO-FS	09	08	32
SSO-FS	13	16	41
KHO-FS	15	18	46
GWO-FS	17	20	52

**Table 4:** Performance Evaluation of Distinct Runs on Proposed AICCP-TBM Method

Dataset-1				
Training Size (%)	Sensitivity	Specificity	Accuracy	F-Score
K = 40	95.62	97.02	97.20	97.60
K = 50	97.00	96.44	97.87	98.40
K = 60	96.81	98.00	97.78	98.06
K = 70	96.90	97.22	97.21	96.76
K = 80	97.04	97.99	96.19	97.25
**Average**	**96.67**	**97.33**	**97.25**	**97.61**

**Dataset-2**				
**Number of Runs**	**Sensitivity**	**Specificity**	**Accuracy**	**F-Score**

K = 40	96.61	98.09	97.81	97.88
K = 50	96.71	97.96	98.05	97.73
K = 60	97.18	96.72	97.54	97.19
K = 70	97.12	98.20	97.45	98.63
K = 80	96.97	97.53	97.63	97.07
**Average**	**96.92**	**97.70**	**97.70**	**97.70**

**Dataset-3**				
**Number of Runs**	**Sensitivity**	**Specificity**	**Accuracy**	**F-Score**

K = 40	96.28	95.31	94.47	94.42
K = 50	95.81	94.40	96.57	93.21
K = 60	97.01	93.95	96.36	92.40
K = 70	94.94	95.31	92.34	93.21
K = 80	95.38	94.86	91.92	93.20
**Average**	**95.88**	**94.77**	**94.33**	**93.29**

**Table 5:** Contrast with Current with Future AICCP-TBM Process for Practical Dataset with admiration to Correctness and F-Score

Methods	Dataset-1		Dataset-2		Dataset-3	
	F-Measure	Accuracy	F-Measure	Accuracy	F-Measure	Accuracy
AICCP-TBM	97.63	97.24	97.72	97.71	93.28	94.34
SSO-OFRBC	96.87	96.97	96.57	96.55	91.77	92.12
SSO-OFRBC	95.45	95.16	95.62	95.10	90.98	91.77
ISMOTE-OWELM	93.52	94.04	91.81	92.01	90.81	90.91
SMOTE-OWELM	93.03	92.21	91.73	91.82	89.91	89.92
OWELM	90.42	90.61	89.42	89.74	87.91	88.72
WELM	88.23	88.52	87.24	87.62	85.22	86.94
PCPM	83.84	83.74	83.15	82.85	80.81	81.83
SVM	76.35	78.93	73.11	72.54	68.22	67.96
LDT/UDT	56.38	85.42	66.23	78.01	59.21	58.04

## Data Availability

The manuscript contains all of the data.
